# Bouveret’s Syndrome as a Rare Life-Threatening Complication of Gallstone Disease—A Surgical Problem: Two Case Reports

**DOI:** 10.3390/medicina61010005

**Published:** 2024-12-24

**Authors:** Nebojsa S. Ignjatovic, Ilija D. Golubovic, Miodrag N. Djordjevic, Marko M. Stojanovic, Daniela A. Benedeto Stojanov, Jelena S. Ignjatovic, Jelena D. Zivadinovic, Sonja Golubovic

**Affiliations:** 1 Clinic for Digestive Surgery, University Clinical Centre Nis, 18000 Nis, Serbia; golubovicilija@yahoo.com; 2 Medical Faculty, University of Nis, 18000 Nis, Serbia; mija.djordjevic@yahoo.com (M.N.D.); marcss994@gmail.com (M.M.S.); dbenedetostojanov@gmail.com (D.A.B.S.); j.ignjat@gmail.com (J.S.I.); jelena5491@gmail.com (J.D.Z.); 3 Clinic for Gastreoenterology, University Clinical Centre Nis, 18000 Nis, Serbia; 4 Clinic for Radiolology, University Clinical Centre Nis, 18000 Nis, Serbia

**Keywords:** Bouveret’s syndrome, gallstone ileus, cholecystectomy, surgical strategy, duodeno-biliary fistula

## Abstract

*Introduction*: Bouveret syndrome, a rare and often underdiagnosed variant of gallstone ileus, is characterized by the presence of a large gallstone impacted in the proximal duodenum, resulting in significant gastric outlet obstruction and aerobilia. Early identification of Bouveret syndrome is crucial for developing an appropriate surgical strategy. *Case 1*: A 76-year-old female underwent a contrast-enhanced abdominal CT scan, which revealed a cholecysto-duodenal fistula with a 3.9 cm × 4.0 cm × 4.0 cm gallstone located in the proximal duodenum, along with a distended, fluid-filled stomach and aerobilia. Intraoperatively, due to chronic inflammation and adhesion between the gallbladder and duodenum, a cholecystectomy and fistula repair were performed. *Case 2*: A 72-year-old female presented with a gastroduodenal passage obstruction confirmed by imaging, which identified a duodeno-biliary fistula. The radiological examination showed oval filling defects in the duodenal bulb consistent with Bouveret’s syndrome, with the largest stone measuring approximately 6 cm in diameter. An enterotomy was performed for stone extraction and was followed by cholecystectomy and duodenal repair with omentoplasty. *Conclusions*: Bouveret’s syndrome is a rare but clinically significant condition that should be considered in patients presenting with signs of upper gastrointestinal obstruction, particularly in those with a history of chronic cholelithiasis. Early recognition and prompt surgical intervention are essential for obtaining optimal patient outcomes.

## 1. Introduction

Bouveret’s syndrome is a rare variant of gallstone ileus resulting from the formation of an acquired fistula between the gallbladder and the stomach or duodenum. When a gallstone passes through this fistula into the enteric system, it can cause gastric outlet obstruction [1]. Biliary ileus is an uncommon complication of cholelithiasis, with only 0.3% to 0.5% of patients with gallstones developing this condition. Additionally, gallstone ileus accounts for approximately 1% to 4% of cases of mechanical intestinal obstruction [2].

The clinical signs indicative of duodenal obstruction, such as a dilated stomach, presence of aerobilia, and the identification of a cholecysto-duodenal fistula, are key to the diagnosis. Further diagnostic imaging, including abdominal ultrasonography, plain abdominal radiography to visualize the gallstone, or contrast-enhanced computed tomography (CT) of the abdomen, can aid in confirming the diagnosis of Bouveret’s syndrome, a rare and life-threatening complication of gallstones [3]. Surgical intervention, either laparoscopic or open, is often required and typically involves enterotomy with stone extraction and primary repair of the enterotomy [4].

This report presents two cases of Bouveret’s syndrome, highlighting distinctive radiological findings, individualized management strategies, and tailored surgical approaches based on the specific conditions of the patients and intraoperative observations. The aim of this paper is to propose a surgical strategy for optimized, patient-specific treatment of suspected Bouveret’s syndrome, drawing on the available literature and our own clinical experience.

## 2. Case Description

This study included two patients, aged 76 and 72, who presented to the emergency department at the Clinic for General Surgery, University Clinical Center Nis. The study was approved by the Institutional Review Board and Human Ethics Committee of the University Clinical Center Nis, Serbia (Approval No. 4258/9). Following detailed anamnesis, both patients underwent laboratory diagnostics. Subsequently, they were evaluated using plain abdominal radiography, abdominal ultrasonography, esophagogastroduodenoscopy (EGD), gastroduodenal passage imaging, and contrast-enhanced abdominal CT scans. Upon completion of the diagnostic workup, both patients were taken to the operating theatre and underwent exploratory laparotomy.

Case 1: A 76-year-old female patient presented with a 3-day history of right upper quadrant pain, nausea, and bilious vomiting. She did not report hematemesis, melena, or any other significant comorbidities. The patient had a known history of gallstones and chronic cholecystitis. On physical examination, she exhibited tenderness to palpation in the right upper quadrant and epigastrium.

Initial laboratory tests revealed only slight abnormalities, as follows: white blood cell (WBC) count of 19 (×10^9^/L), neutrophil percentage (Neu) of 91.1%, C-reactive protein (CRP) of 92 mg/L, blood glucose (Glu) of 124 mg/dL, urea of 76.1 mg/dL, creatinine (Crea) of 1.06 mg/dL, γ-glutamyl transferase (γGT) of 101 mg/dL, total bilirubin (tBIL) of 1.42 mg/dL, direct bilirubin (dBIL) of 1.13 mg/dL, lactate dehydrogenase (LDH) of 554 U/L, sodium (Na) of 137 mEq/L, potassium (K) of 4 mEq/L, hemoglobin (Hb) of 13 g/dL, and a platelet count of 350,000 cells/μL.

**Figure 1:**
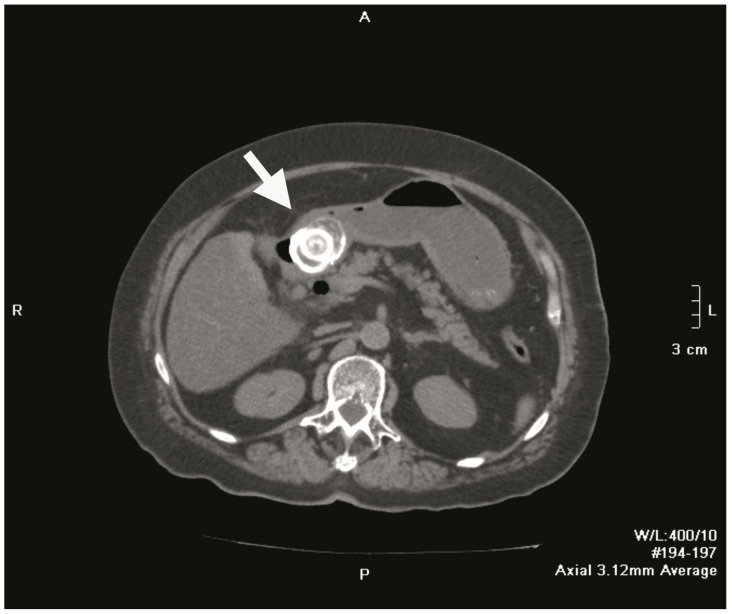
Axial non-contrast CT image of the upper abdomen revealing a large gallstone (white arrow) lodged in the duodenal bulb, a finding characteristic of Bouveret’s syndrome; the gallstone is obstructing gastric outflow and causing marked gastric distention. An air-fluid level is observed within the distended stomach.

**Figure 2:**
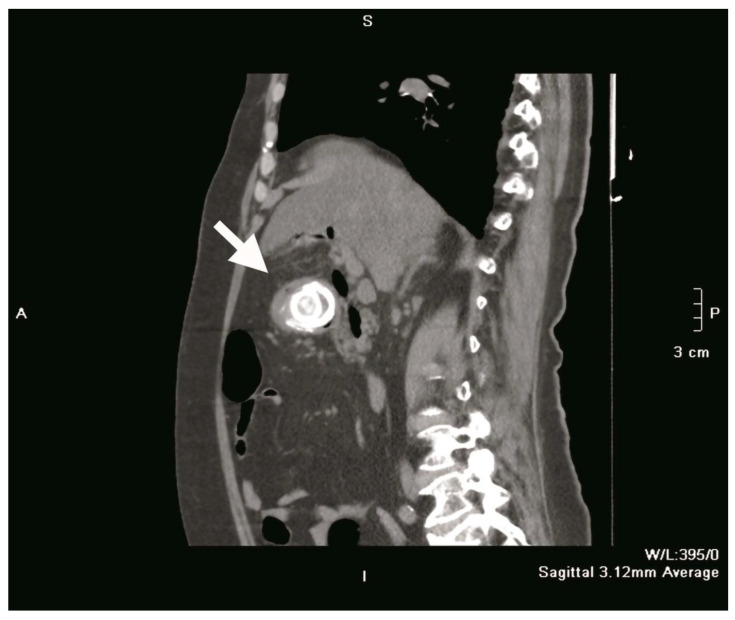
Sagittal section CT scan showing a gallstone (white arrow) impacted in the duodenal bulb.

Plain abdominal radiography revealed a distended, fluid-filled stomach with signs of pneumoperitoneum (a result of gallbladder perforation; however, there was no secondary peritonitis because the process was blocked by the greater omentum) and obstruction. A 4 cm shadow of calcium intensity was noted in the right lumbar region. Abdominal ultrasonography demonstrated a partially distended, thick-walled gallbladder with multiple calculi, a finding consistent with chronic cholecystitis. Follow-up contrast-enhanced abdominal CT confirmed the presence of a cholecysto-duodenal fistula in the first part of the duodenum, with a 3.9 cm × 4.0 cm × 4.0 cm gallstone lodged in the proximal duodenum (Figure 1 and Figure 2). The scan also revealed sludge and calculi in the gallbladder, along with a distended, fluid-filled stomach and aerobilia.

EGD was performed and identified a 4 cm gallstone in the first part of the duodenum. The clinical presentation and imaging findings were consistent with Bouveret’s syndrome and were characterized by a cholecysto-duodenal fistula and the impaction of a large gallstone in the duodenal bulb.

**Figure 3:**
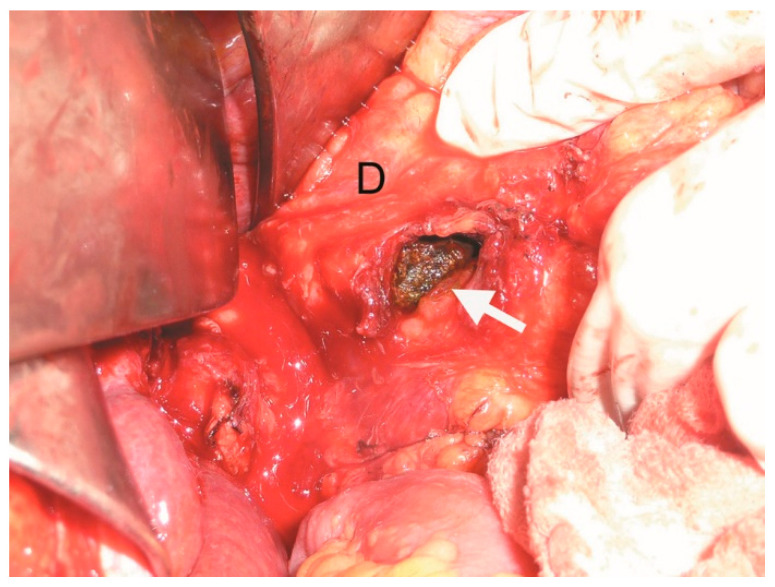
Intraoperative image showing duodenotomy (white arrow) with the impacted gallstone (D—duodenum).

**Figure 4:**
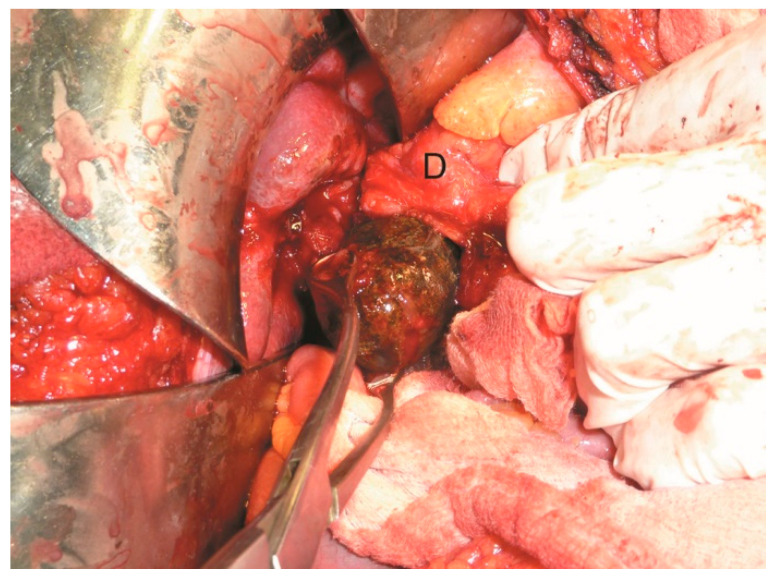
Intraoperative image showing the extraction of the impacted gallstone (D—duodenum).

**Figure 5:**
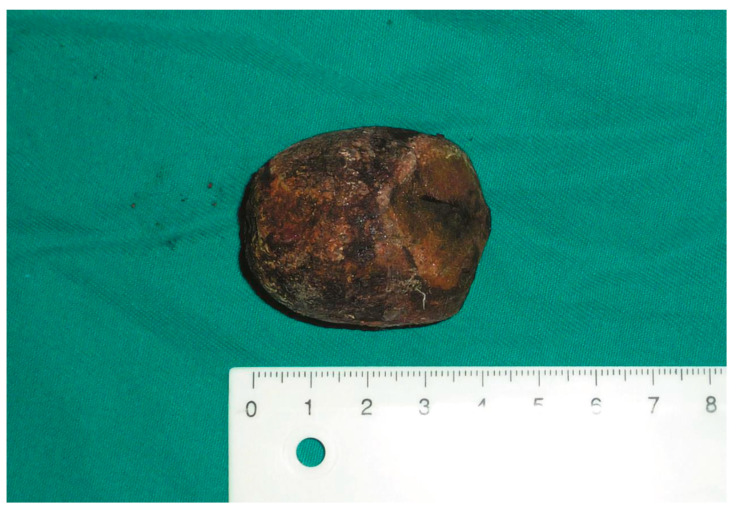
The removed gallstone.

The patient was then taken to the operating theatre for exploratory laparotomy. Intraoperatively, significant chronic inflammation and adhesion between the gallbladder and the duodenum were noted. A cholecystectomy and repair of the cholecysto-duodenal fistula were performed through the orifice of the fistula corresponding to the duodenum. The duodenum was closed with separate sutures in two layers with synthetic absorbable suture (3-0 Vicryl^®^, Ethicon Inc., Raritan, NJ, USA). The small bowel distal to the stone was not dilated. The gallstone fragment removed from the duodenum measured 3.9 cm × 4.0 cm × 4.0 cm (Figure 3, Figure 4 and Figure 5).

Postoperatively, the patient was transferred to the intensive care unit for 24 h and then moved to the general surgical ward. There were no intraoperative or postoperative complications. A naso-enteral tube was present for 6 days. The patient underwent a period of intensive rehabilitation, which included physiotherapy, total parenteral nutrition, and a gradual reintroduction of oral feeding. She remained hospitalized for 10 days postoperatively, after which she was discharged in stable condition, when she had been weaned off TPN and was tolerating a full oral diet. The patient had an uneventful recovery and remains in good health at the time of writing.

Case Report 2: A 72-year-old female patient was admitted with a 3-day history of non-specific symptoms, including loss of appetite, nausea, vomiting of watery brownish content, epigastric pain, abdominal distension, and constipation. She had a longstanding history of gallstones and chronic cholecystitis. On examination, the patient appeared to be in poor general condition, subfebrile, eupneic, and tachycardic. Active movements were difficult for her due to discomfort. Palpation of the abdomen revealed a soft consistency with mild epigastric tenderness, but no peritoneal signs were present. The “churning phenomenon” was positive.

Laboratory findings showed a WBC count of 15 (×10^9^/L), neutrophils (Neu) 89.3%, C-reactive protein (CRP) of 85 mg/L, blood glucose (Glu) of 141 mg/dL, urea of 81.1 mg/dL, creatinine (Crea) of 1.23 mg/dL, γ-glutamyl transferase (γGT) of 91 mg/dL, total bilirubin (tBIL) of 1.42 mg/dL, direct bilirubin (dBIL) of 1.13 mg/dL, lactate dehydrogenase (LDH) of 554 U/L, sodium (Na) of 138 mEq/L, potassium (K) of 4.1 mEq/L, hemoglobin (Hb) of 14.8 g/dL, and a platelet count of 380,000 cells/μL.

**Figure 6:**
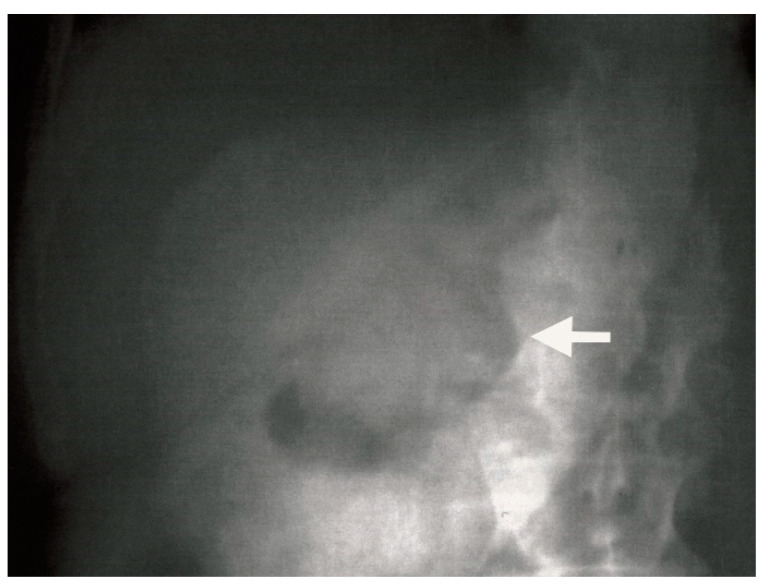
Plain abdominal radiograph showing pneumobilia (white arrow) in the gallbladder.

Ultrasound examination of the abdomen revealed a collapsed gallbladder with multiple gallstones of varying sizes, the largest measuring 4.2 cm. This raised concern for possible perforation of the gallbladder. Plain abdominal radiography revealed the presence of gas in the bile ducts, or aerobilia (Figure 6).

**Figure 7:**
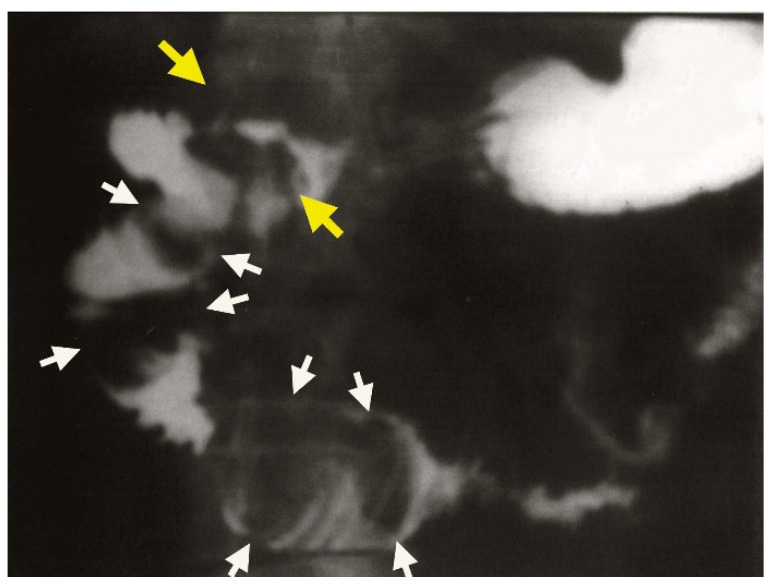
Gastroduodenal passage showing a cholecystoduodenal fistula (yellow arrow) with impacted stones in the duodenum. Oval filling defects are clearly delineated in the duodenal bulb (white arrows).

**Figure 8:**
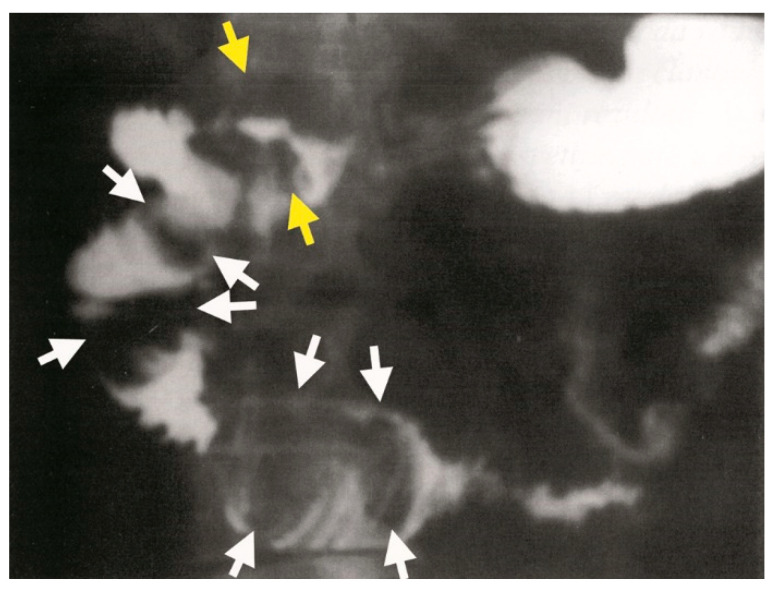
Gastroduodenal passage showing multiple oval filling defects clearly localized in the duodenal bulb (yellow arrows) and extending through the D1, D2, and D3-D4 segments of the duodenum (white arrows).

A proximal endoscopy identified a 2 cm gallstone in the duodenal bulb, which appeared yellowish-green in color. The mucosa of the posterior wall of the bulb was eroded and hyperemic, but there was no evidence of a wall defect. Further diagnostic evaluation through gastroduodenal passage imaging confirmed the presence of a duodeno-biliary fistula (Figure 7). In the duodenal bulb, oval filling defects consistent with Bouveret’s syndrome were observed, with the largest stone measuring approximately 6 cm in diameter (Figure 8).

The patient was subsequently taken to the operating theatre, where an exploratory laparotomy was performed. An enterotomy was conducted to extract the stones and was followed by cholecystectomy and duodenal suturing with separate sutures in two layers (synthetic absorbable suture, 3-0 Vicryl^®^), as well as with omentoplasty.

Postoperatively, the patient was admitted to the intensive care unit for 24 h, after which she was transferred to the general surgical ward. There were no intraoperative or postoperative complications. The patient underwent an intensive rehabilitation program, including physiotherapy, total parenteral nutrition, and gradual reintroduction of an oral diet. She was hospitalized for 8 days postoperatively and was discharged after being weaned off TPN, when she was tolerating a full oral diet. The patient had an uneventful recovery and remains in good health at the time of writing.

## 3. Discussion

Bouveret’s syndrome was first described by Leon Bouveret in 1896, who presented two cases of patients with this condition [5]. He was the first to describe the pathophysiology of a large gallstone obstructing the duodenal bulb after having passed through a cholecysto- or choledochoduodenal fistula, finally resulting in gastric outlet obstruction, in a condition now recognized as Bouveret’s syndrome [6]. While gallstones typically cause obstruction in the distal ileum (60–70%), they can also be found in the proximal ileum (25%), distal ileum (10%), jejunum (9%), colon (4%), and rectum (2%). However, gallstones impacting the duodenum represent a rare occurrence, with an incidence of only 1–3% [7], as highlighted in the present case report. In some cases, gallstones may enlarge while traversing the fistula and the intestine due to the accumulation of fecal material and salts on their surface [8]. Bouveret’s syndrome is a rare cause of gastric outlet obstruction wherein a large gallstone passes through a bilioenteric fistula, causing duodenal obstruction [9].

Bouveret’s syndrome is believed to result from recurrent episodes of cholecystitis, which lead to the formation of adhesions between the gallbladder and adjacent parts of the upper gastrointestinal tract. The continuous pressure exerted by a large stone causes necrosis and perforation of the gallbladder wall, leading to the formation of a fistula with the duodenum or stomach [10].

Risk factors for the development of Bouveret’s syndrome include advanced age (over 70 years), gallstones larger than 2.5 cm, female gender, and post-surgical alterations to the gastrointestinal anatomy [5,7]. In our report, both patients were female and aged over 70 years (76 and 72 years), with 4 cm gallstones and histories of chronic cholecystitis.

Key factors contributing to perforation include the size of the gallstone, the size of the bowel lumen, and the site of fistula formation [11]. Stones smaller than 2.5 cm typically pass through the small bowel smoothly or result in gallstone ileus, a more common occurrence with larger stones [12]. In this report, duodenal obstruction was caused by a 4 cm gallstone, which is large enough to cause significant complications requiring urgent intervention [13].

The mortality rate for gallstone ileus is reported to range from 7% to 30% (average 18%). This high mortality is primarily due to factors such as advanced age, frailty, multiple comorbidities (particularly cardiovascular, respiratory, and endocrine conditions like diabetes and obesity), and delayed presentation (typically 4–8 days after symptom onset). The literature suggests a median delay of 2–37 days between admission and surgical intervention, with a range of 1–15 days [14].

The clinical presentation of Bouveret’s syndrome is often nonspecific and infrequent, leading to delayed diagnosis [15]. Symptoms can range from gastric outlet obstruction (as seen in our patients) to acute pancreatitis, upper gastrointestinal bleeding, duodenal perforation, Boerhaave’s syndrome, and gastric bezoar formation [16]. Ileus is not typically a sign of the presence of a gallstone. The majority of the patients experience symptoms that include nausea, vomiting, and epigastric pain [8]. In our case, both patients exhibited symptoms of nausea, bilious vomiting, and epigastric pain for three consecutive days.

The choice of diagnostic approach is critical in achieving a timely and accurate diagnosis of Bouveret’s syndrome. It remains a diagnostic challenge, with approximately 50% of diagnoses confirmed only during surgical intervention [8]. Although EGD may lead to a more definite diagnosis in Bouveret’s syndrome, a direct abdominal X-ray is still the first step in the approach to these patients, as it allows surgery to be performed as early as possible [15].

EGD is crucial both diagnostically and therapeutically, as it allows visualization of the dilated stomach and impacted gallstone, which typically appears as a firm, non-fleshy mass. EGD can also reveal the duodenal ostium of the biliodigestive fistula [17]. An upper gastrointestinal series with oral contrast may provide additional insight into the obstructing mass by showing a filling defect, a gallstone, dilation of the stomach or duodenum, pneumobilia, and/or outlet obstruction. In rare cases, contrast extravasation into the gallbladder can indicate a patent cholecystoduodenal or cholecystogastric fistula [15]. In our cases, initial endoscopy confirmed the presence of the stone and the obstruction.

Imaging studies, particularly contrast-enhanced CT scans, are pivotal in diagnosing Bouveret’s syndrome. The diagnosis typically begins with a plain abdominal X-ray, though this is diagnostic in only 21% of cases [8]. In our report, the X-ray showed a 4 cm radiopaque shadow in the right lumbar region, suggesting the presence of a gallstone without signs of ileus or pneumoperitoneum.

Radiologically, Bouveret’s syndrome is often identified by “Rigler’s triad”, which is seen in approximately 30–35% of cases [7,16]. The classic Rigler’s triad—dilated stomach, pneumobilia, and an ectopic stone seen as a filling defect in the duodenum on CT—is considered virtually pathognomonic of Bouveret’s syndrome [16].

In 60% of cases, abdominal ultrasonography can be helpful, revealing an ectopic gallstone, a fluid-filled distended stomach, pneumobilia, and features indicative of cholecystitis [7,8]. However, its limitations include difficulty in locating the stone and interference from excessive intestinal gas. Gastroduodenal passage imaging, which is useful in approximately 45% of cases, can help identify the fistula and locate the stone, revealing oval filling defects during the migration of the calculi through the intestinal lumen [12]. In 60% of cases, a CT scan provides definitive diagnostic information and is highly sensitive, specific, and accurate (93%, 100%, and 99%, respectively) [7,13]. In 45% of the cases, as one of the radiological imaging techniques, imaging of the gastroduodenal passage can be helpful in identifying the fistula and locating the stone. Numerous defects of oval fillings can be discovered during the migration of calculi in the lumen of the intestine [12].

In 60% of cases, a CT scan provides definitive diagnostic information, as well as an evaluation of the gallstones, fistulas, and inflammatory findings. It offers 93% sensitivity, 100% specificity, and 99% accuracy [7,13]. This imaging modality is considered the gold standard for diagnosing Rigler’s triad in gallstone ileus [8]. Plain abdominal films show Rigler’s triad in 14.8 to 21% of cases, while the rates of positive findings are 11.1% for ultrasound and 77.8% for CT scan. Although CT scans are highly effective, limitations exist in detecting gallstones with isoattenuation (15–25%), necessitating additional imaging [13]. The impacted gallstone is endoscopically visible in 70% of cases, most likely due to the fact that the mucosa covers the embedded stone. The complete diagnosis is made during surgical procedures in 20–40% of cases [17]. In this case report, the presence of gallstones was confirmed by all imaging diagnostic procedures.

The primary aim in treating Bouveret’s syndrome is to remove the obstructing gallstone. Both nonsurgical (endoscopic) approach and surgical (open or laparoscopic) management are therapeutic options [18,19,20]. Considering the surgical morbidity and given the fact that most patients are elderly, with multiple comorbidities, the endoscopic approach should be the first line of treatment when it comes to Bouveret’s syndrome. However, it is complicated to dislodge and remove a large, impacted stone. Thus, the endoscopic approach is not very successful and is rarely therapeutic [13]. Moreover, some researchers feel that endoscopic lithotomy may increase the risk of esophageal injury, digestive tract perforation, and gastrointestinal bleeding. Research showed that 42% of cases did not end in stone removal by this technique [8].

Surgical intervention is necessary in more than 91% of cases. Depending on the patient state, the surgical procedure can be one-staged enterolithotomy (or gastrotomy) with concomitant cholecystectomy and repair of the fistula or enterolithotomy alone with or without a second-stage cholecystectomy [13]. Recently, with the extensive development and use of laparoscopy, laparoscopic surgery for Bouveret’s syndrome has shown to be a safe and effective alternative to open surgery [8].

The optimal surgical approach should be individualized based on the patient’s age, general and local health, comorbidities, and life expectancy and the size and location of the stone and fistula [17]. In these cases, taking into account the presence of the large, impacted gallstone in the duodenum, the endoscopic approach is not the best option.

The surgical procedure involved open enterolithotomy with concomitant cholecystectomy and repair of the cholecystoduodenal fistula.

Bouveret’s syndrome is associated with significant morbidity and mortality [15]. Gallstone ileus, in general, carries a poor prognosis, particularly in the elderly. Mortality and morbidity rates have decreased over time (from 30% to 12%), yet they remain high, primarily due to delayed diagnosis and concurrent medical conditions [13,18]. As a rare complication resulting from the large-scale impaction of a gallstone in the duodenal bulb and subsequent gastric outlet obstruction, Bouveret’s syndrome requires prompt and accurate diagnosis, as well as early surgical intervention, to optimize outcomes. However, due to the small sample size, more extensive research is not possible, and, consequently, accurate results should be derived from a better statistical sample. The selected strategy and approach should be patient-specific and based on characteristics such as age, general and local status, medical condition of the patient in relation to the morbidity, and the mortality rates of each approach.

## 4. Conclusions

Bouveret’s syndrome is a rare but critical clinical condition that should be considered in patients presenting with ileus in the upper abdomen, particularly those with a history of chronic cholelithiasis. Its symptoms are nonspecific and can be life-threatening, especially in elderly patients. Early and accurate diagnosis, followed by timely surgical intervention, is essential for effective treatment. Although endoscopy may be a useful diagnostic and occasionally therapeutic tool, surgical management is often necessary, particularly when endoscopic approaches fail or are not available. The surgical procedure can be one-staged enterolithotomy (or gastrotomy) with concomitant cholecystectomy and repair of the fistula or enterolithotomy alone with or without a second-stage cholecystectomy. The surgical strategy should be individualized, taking into account the patient’s overall health and comorbidities and the specific characteristics of the gallstone and fistula. Bouveret’s syndrome, as the appropriate treatment, can pose a challenge for the surgeon when surgery is needed.

## Data Availability

Data is contained within the article.

## Abbreviations

CT: Computed tomography; CM: Centimeter; WBC: White blood cell count; Neu: Neutrophils; Glu: Glucose; Crea: Creatinine; γGT: Gamma-glutamyl transferase; tBIL: Total bilirubin; dBIL: Direct bilirubin; LDH: Lactate dehydrogenase.
